# Granulomatosis With Polyangiitis: A Pauci-Immune Rapidly Progressive Glomerulonephritis With Isolated Renal Involvement in an Elderly Male

**DOI:** 10.7759/cureus.17098

**Published:** 2021-08-11

**Authors:** Wasey Ali Yadullahi Mir, Dhan B Shrestha, Vijay K Reddy, Anurag Adhikari, Larissa Verda

**Affiliations:** 1 Internal Medicine, Mount Sinai Hospital, Chicago, USA; 2 Medicine, Mount Sinai Hospital, Chicago, USA; 3 Intensive Care Unit, Nepal Korea Friendship Municipality Hospital, Madhyapur Thimi, NPL

**Keywords:** wegener’s granulomatosis, antineutrophil cytoplasmic antibodies, granulomatosis with polyangiitis, glomerulonephritis, abdominal pain

## Abstract

Granulomatosis with polyangiitis (GPA) is a necrotizing vasculitis with upper and lower respiratory tract and renal system involvement. We present a case of a 59-year-old male presenting with complaints of abdominal pain with deranged renal function and acute increase in creatinine level. On investigation, the antineutrophil cytoplasmic autoantibody, cytoplasmic (c-ANCA) was found to be significantly elevated in association with pauci-immune crescentic glomerulonephritis on biopsy. This was diagnostic of Wegener’s granulomatosis. He was treated with intravenous cyclophosphamide 10 mg/kg/pulse along with steroids at 1 mg/kg/day for induction and trimethoprim/sulfamethoxazole (TMP-SMX) 80/400 mg for pneumocystis carinii pneumonia (PCP) prophylaxis after a negative tuberculosis QuantiFERON® assay (Qiagen, Netherlands). On discharge, he was on TMP-SMX prophylaxis for PCP, prednisone 60 mg daily, and cyclophosphamide on pulse dosing every 14 days with instructions to follow up. The patient showed improvement in therapy.

## Introduction

Granulomatosis with polyangiitis (GPA), also known as Wegener’s granulomatosis, is an antineutrophil cytoplasmic antibody (ANCA)-associated small vessel vasculitis. It is a multisystem disorder affecting the upper and lower respiratory tracts, and the renal system [1]. It is characterized by necrotizing vasculitis of the respiratory tract and often involves the renal system late in the course of the disease with focal or segmental glomerulonephritis [2]. This report presents a case of granulomatosis with polyangiitis, with cytoplasmic-ANCA (c-ANCA)-positive rapidly progressive glomerulonephritis (RPGN) evident on immunofluorescence and electron microscopic reading of renal biopsy specimen.

## Case presentation

A 59-year-old Hispanic male with a history of bilateral knee osteoarthritis presented with complaints of abdominal pain located in the epigastric and right upper quadrant. The onset of pain was sudden, and it was moderate in intensity. The patient had the pain for the last three days, and it was intermittent and colicky. The pain was associated with constipation and abdominal bloating since its inception. He did not report fever or vomiting. The patient had recently taken ibuprofen for his pain but stopped since he developed excessive sweating after the third dose.

Laboratory analysis showed hemoglobin of 8.8 gm%, mean cell volume (MCV) of 77 femtoliters, ferritin of 898 ng/mL, and a WBC count of 12.4*10^9/L cells with neutrophil 88% without band cells. The notable rise in acute phase reactants was seen with an erythrocyte sedimentation rate (ESR) of >140 mm/h and C-reactive protein (CRP) of 246 mg/L. On admission, his creatinine level was found significantly elevated to 5.04 mg/dL from 3.6 mg/dL noted during his clinic visit a day before admission. His creatinine level was 0.8 mg/dL on his records two years ago. The liver function tests (LFTs) were normal except for the alkaline phosphatase level, raised to 123 IU/L. Other abnormal labs included a total protein of 7.7 g/dL, albumin of 2.7 g/dL, and globulin of 4.1 g/dL (gamma-globulin elevated at 2.1 g/dL). Urinalysis showed cloudy urine with RBC and WBC casts, moderate leukocyte esterase, and an elevated protein-creatinine ratio at 2.88.

**Figure 1:**
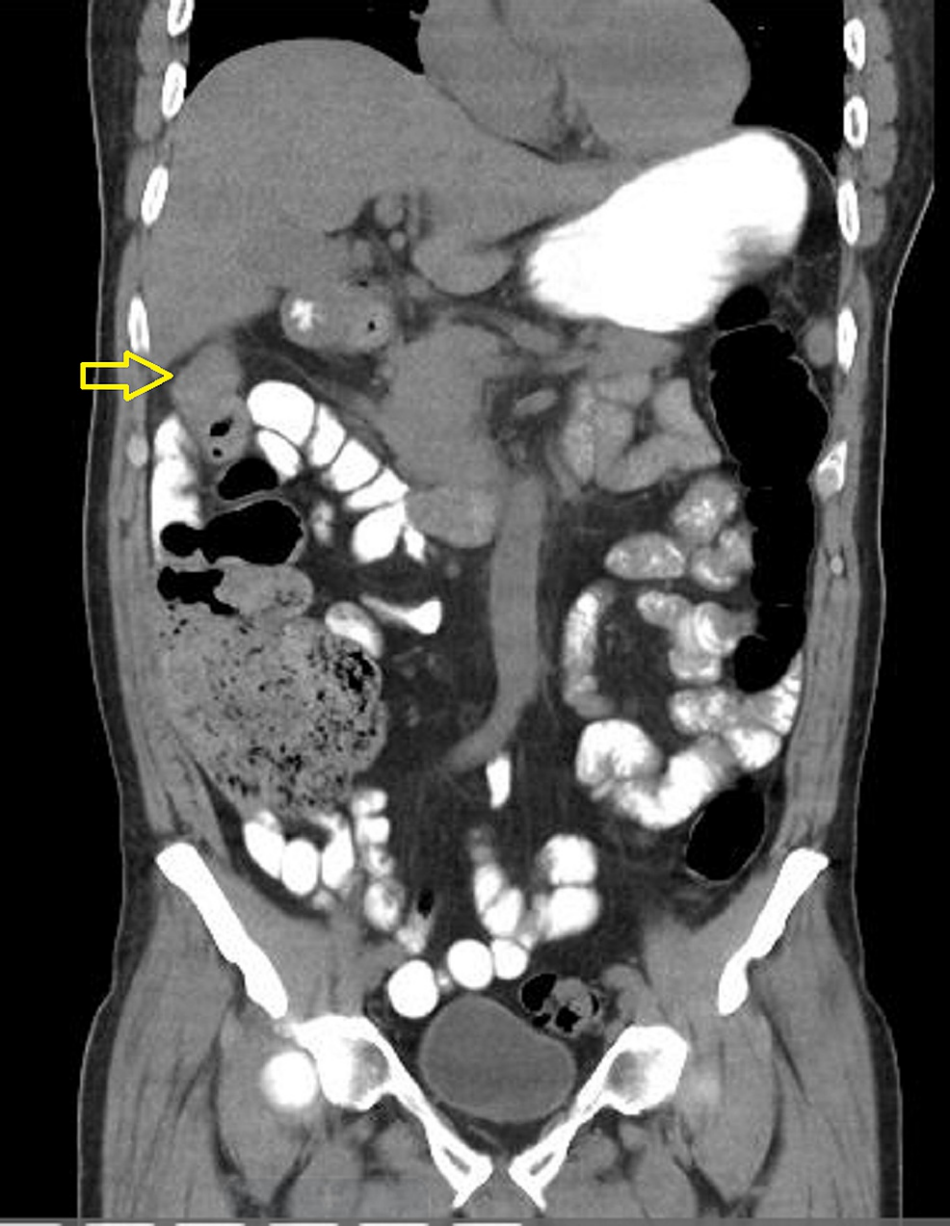
Coronal section of abdominal CT scan showing thickened hepatic flexure of the colon (marked with arrow)

**Figure 2:**
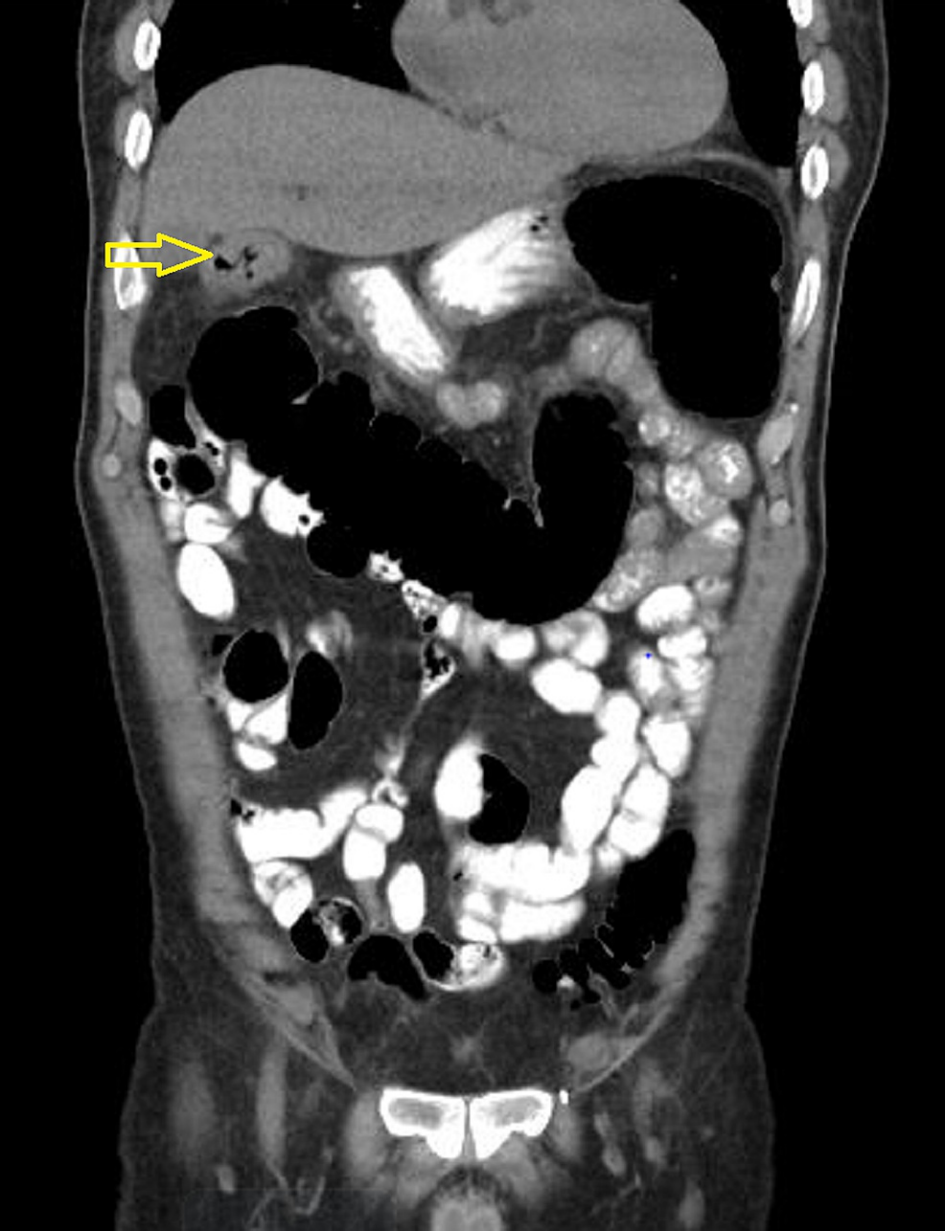
Coronal section of abdominal CT scan showing thickened hepatic flexure of the colon (marked with arrow)

A CT scan of the abdomen and pelvis performed at admission showed segmental wall thickening at the hepatic flexure (Figure 1 and Figure 2). He was started on metronidazole 500mg intravenous eight hourly for possible colitis, and ceftriaxone 1gm twice a day for urinary tract infection (UTI). In addition, rouleaux formation was seen on a peripheral blood smear (PBS); therefore, urine protein electrophoresis (UPEP) and serum protein electrophoresis (SPEP) was also done to rule out multiple myeloma. In addition, C3, C4, anti-streptolysin antibody (ASO), rheumatoid factor (RF), and antinuclear antibody (ANA) were sent for autoimmune workup.

**Figure 3:**
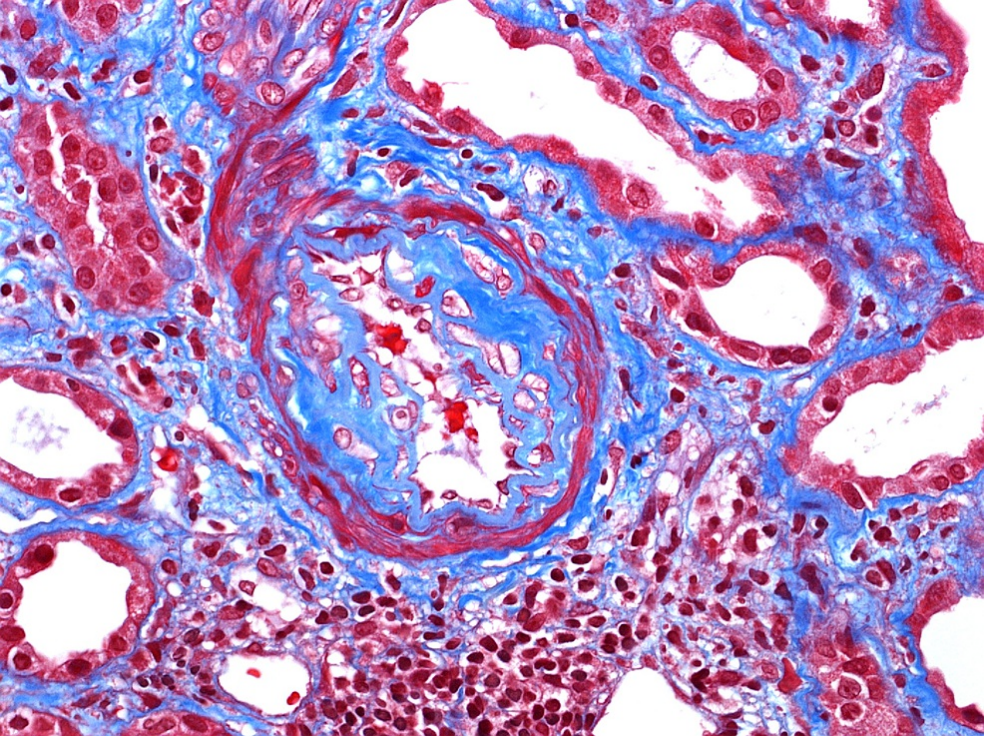
High-resolution histopathological imaging of renal biopsy specimen showing intimal fibrosis of arteriole

**Figure 4:**
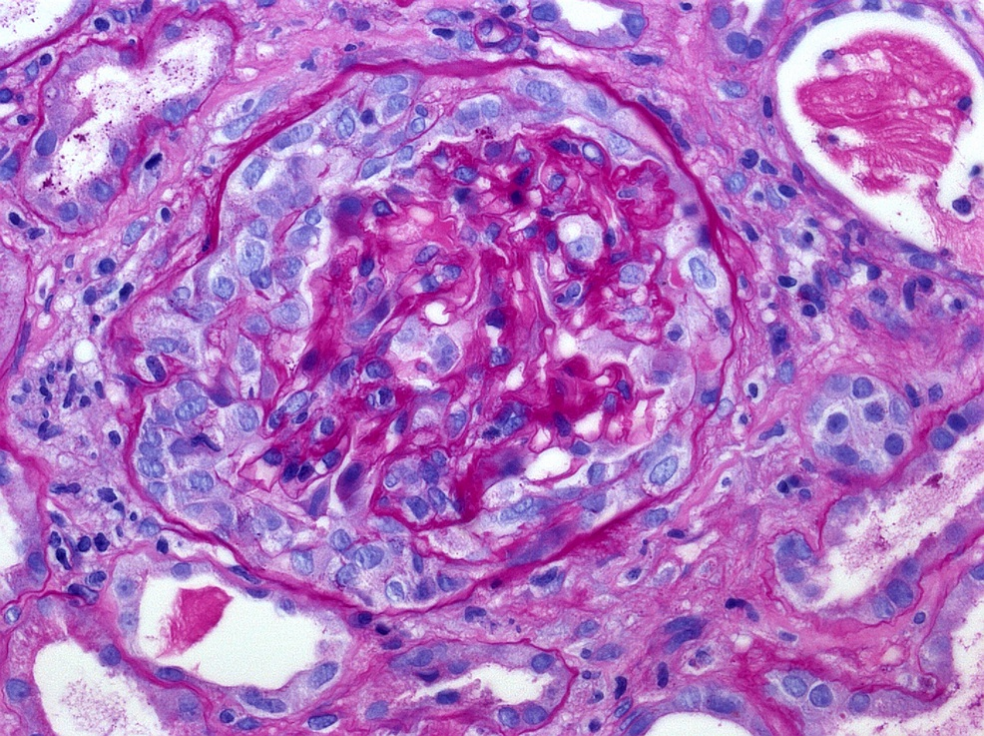
High-resolution imaging of histopathological specimen of renal biopsy showing the glomerular crescent formation

**Figure 5:**
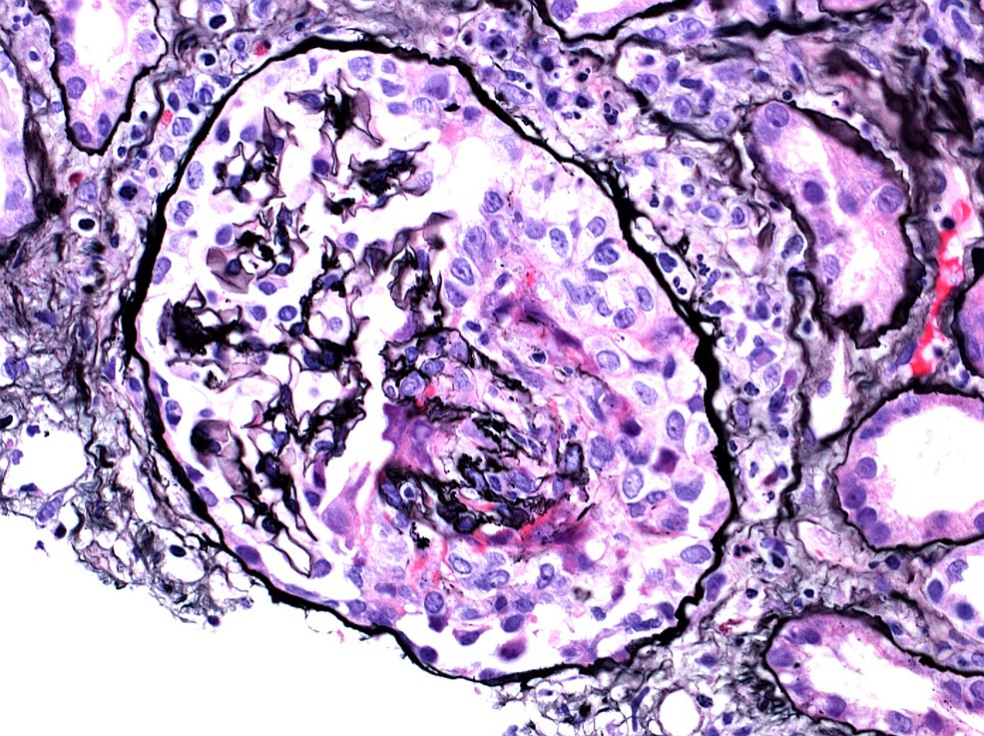
High-resolution histopathological imaging of renal biopsy specimen showing the necrotizing glomerular crescent

**Figure 6:**
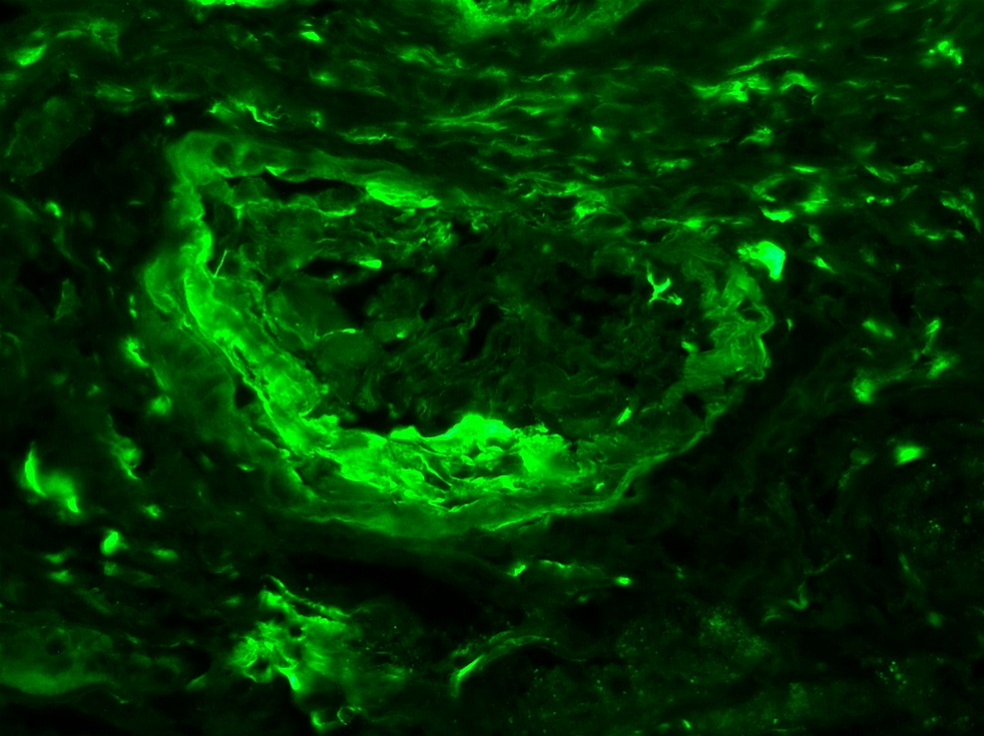
High-resolution immunofluorescence imaging of renal biopsy specimen showing glomerular fibrinogen staining

On the second day of admission, the blood and urine cultures were negative, with a chest x-ray showing normal findings. The patient was negative for hepatitis A, B, C, and HIV. The autoimmune workup was also normal. Myeloperoxidase antibody (MPO Ab) perinuclear-ANCA (p-ANCA) and proteinase 3 antibody (PR3) c-ANCA tests were then ordered. On the fourth day of admission, renal USG was performed, which did not show any obstruction. The abdominal pain resolved on day five of admission, and a renal biopsy was performed. It showed RPGN from pauci-immune vasculitis (Figure 3, 4, 5, 6). Electrophoretic studies showed polyclonal elevation but no monoclonal bands. He was started on methylprednisolone 1gm for three days for RPGN on day six of admission. The c-ANCA level was found to be positive at 55.4 U/mL (reference range <1 - no antibody, > 1 - antibody detected) with a p-ANCA <1 U/mL. He was started on intravenous cyclophosphamide 10mg/kg/pulse every two weeks and prednisone 1mg/kg/day for induction and trimethoprim-sulfamethoxazole(TMP-SMX) 80/400 mg for pneumocystis carinii pneumonia prophylaxis after a negative tuberculosis QuantiFERON® assay (Qiagen, Netherlands). With this therapy, the level of creatinine improved from 5.81 on day six to 4.03 on day nine.

He was discharged on TMP-SMX prophylaxis for PCP, prednisone 1mg/kg/day, and cyclophosphamide 10mg/kg/pulse every 14 days with instructions to follow up. On follow-up, ten days later, creatinine had improved to 2.5 mg/dL.

## Discussion

A necrotizing vasculitis, GPA is characterized by granulomatous inflammation of upper and lower respiratory tracts and glomerulonephritis. Lung involvement shows necrotizing vasculitis, which is the most frequently involved organ system by GPA. It may manifest as multiple nodules and masses in the lung parenchyma [3]. Renal involvement is usually manifested as focal or segmental glomerulonephritis. However, sometimes it may manifest as a renal mass which is a rare finding in GPA [2].

Autoantibodies directed against proteinase 3 (c-ANCA), a constituent of neutrophil azurophilic granules, are a strong indicator of the disease, which is deemed a danger signal for the induction of autoimmune inflammation. The diagnosis is confirmed by the tissue finding of necrotizing vasculitis [4]. However, ANCA is not required to make a diagnosis of GPA by either the American College of Rheumatology (ACR) or the Chapel Hill Consensus Conference (CHCC) definitions [5,6]. Other types of ANCA (p-ANCA, a-ANCA, x-ANCA) are not specific to GPA and can be elevated in various inflammatory conditions [7]. Hoffman and colleagues (1992) reviewed 158 patients over 24 years in the National Institute of Health (NIH). The presenting symptom in 90% of the cases was respiratory with involvement of lower and upper airways [8]. Lung involvement at presentation was seen in 45% of the patients and cutaneous involvement in 25% of the cases. In this study, only 18% of the patients presented with renal disease.

The histopathological findings can range from mild focal segmental glomerulonephritis with minimal renal impairment to diffuse necrotizing glomerulonephritis with fulminant proliferative and crescentic changes in renal diseases. Renal disease is invariably associated with other systemic findings; therefore, our case with isolated renal involvement is unique in presentation and progression. Renal disease in GPA, when present, progresses from mild to severe form within days to weeks [9]. The patient may deteriorate even with treatment.

Our patient presented with the complaint of abdominal pain in the epigastric and right upper quadrant region. His vital signs were stable throughout the hospital stay except for an isolated episode of fever with a temperature of 38.1°C. As the patient had a history of gastritis, a possible diagnosis of *Helicobacter pylori*-related gastritis was made. The patient was started on a triple regimen of metronidazole, clarithromycin, and pantoprazole. On CT of the abdomen and pelvis, thickening of the bowel wall was noted on hepatic flexure. Therefore, colitis was also a differential diagnosis for which metronidazole was given. Since the renal function was deranged with urine findings of RBC and RBC casts along with WBC and WBC casts with moderate leukocyte esterase, he was started on ceftriaxone for a possible diagnosis of UTI. However, the creatinine level was continually elevated, and further workup for autoimmune diseases and a renal biopsy were performed to rule out Immunoglobulin A (IgA) nephropathy and primary focal segmental glomerulosclerosis (FSGS). Urine and serum protein electrophoresis were performed to rule out multiple myeloma as PBS showed rouleaux formation. c-ANCA was elevated with renal findings suggestive of glomerulonephritis in the presence of negative ANA, p-ANCA, and rheumatoid factor, which was virtually diagnostic for Wegener’s granulomatosis [10].

Our patient presented with an isolated renal finding in the absence of associated respiratory or cutaneous symptoms, which are less likely. The combination of high-dose corticosteroids and cyclophosphamide is the mainstay of treatment for the GPA and vasculitis. The risk of disease resistance to this combination is uncommon. Therefore, intravenous (IV) cyclophosphamide (0.5g/m^2 to 1.0g/m^2 body surface area) and pulse methylprednisolone (1g IV for three days), followed by high-dose steroid is recommended. In addition, cyclophosphamide is repeated at 4-week intervals. The therapy with methylprednisone was started and continued for three days [11]. The treatment with cyclophosphamide is found to be life-saving. Accordingly, our patient was started on intravenous methylprednisolone 1gm for three days and IV cyclophosphamide 100mg for induction. Prolonged treatment with TMP-SMX is postulated to reduce the cumulative dose of immunosuppressant and reduce the relapse rate [12]. Therefore, our patient was discharged on TMP-SMX and prednisolone.

## Conclusions

GPA can present with renal disease; however, an isolated renal finding without multisystem involvement is uncommon. Nevertheless, since the disease can progress rapidly and lead to end-stage renal failure, the patient should be promptly started on high-dose steroid therapy and cyclophosphamide. In addition, TMP-SMX is recommended for PCP prophylaxis to prevent the relapse of GPA.
